# Imaging Findings of Pelvic Tumor Thrombosis Extending from Sacral Bone Metastasis of Adrenocortical Carcinoma

**DOI:** 10.1155/2012/919603

**Published:** 2012-12-26

**Authors:** Kenichiro Ishida, Yusuke Inoue, Reiko Woodhams, Yuji Asano, Toshimasa Hara

**Affiliations:** Department of Diagnostic Radiology, Kitasato University School of Medicine, 1-15-1 Kitasato, Minami-ku, Sagamihara, Kanagawa 252-0373, Japan

## Abstract

We report the imaging findings of a patient with adrenocortical carcinoma who showed pelvic tumor thrombosis extending from sacral bone metastasis. Contrast-enhanced computed tomography demonstrated extensive intraluminal filling defects in the pelvic veins. A lytic lesion in the sacrum was also noted and continuity between the sacral lesion and the filling defect in the branch of pelvic veins was indicated. The filling defects showed increased uptake on positron emission tomography with ^18^F-fluorodeoxyglucose and single-photon emission computed tomography with ^131^I-iodomethylnorcholesterol, and fusion images with computed tomography aided the localization of the increased uptake areas. Multimodality imaging may be beneficial for the characterization and localization of lesions in patients suspected of having metastatic adrenocortical carcinoma.

## 1. Introduction

Correct diagnosis of tumor thrombosis and its differentiation from venous blood thrombosis are important for determining treatment, especially selection from anticoagulation and anticancer treatments. Adrenocortical carcinoma can cause venous tumor thrombosis [1], which may grow extensively [2, 3]. This paper presents computed tomography (CT), positron emission tomography-(PET-) CT with ^18^F-fluorodeoxyglucose (^18^F-FDG), and single photon emission computed tomography-(SPECT-) CT with ^131^I-iodomethylnorcholesterol findings in a patient with adrenocortical carcinoma who showed pelvic tumor thrombosis extending from sacral bone metastasis.

## 2. Case Report

**Figure 1:**
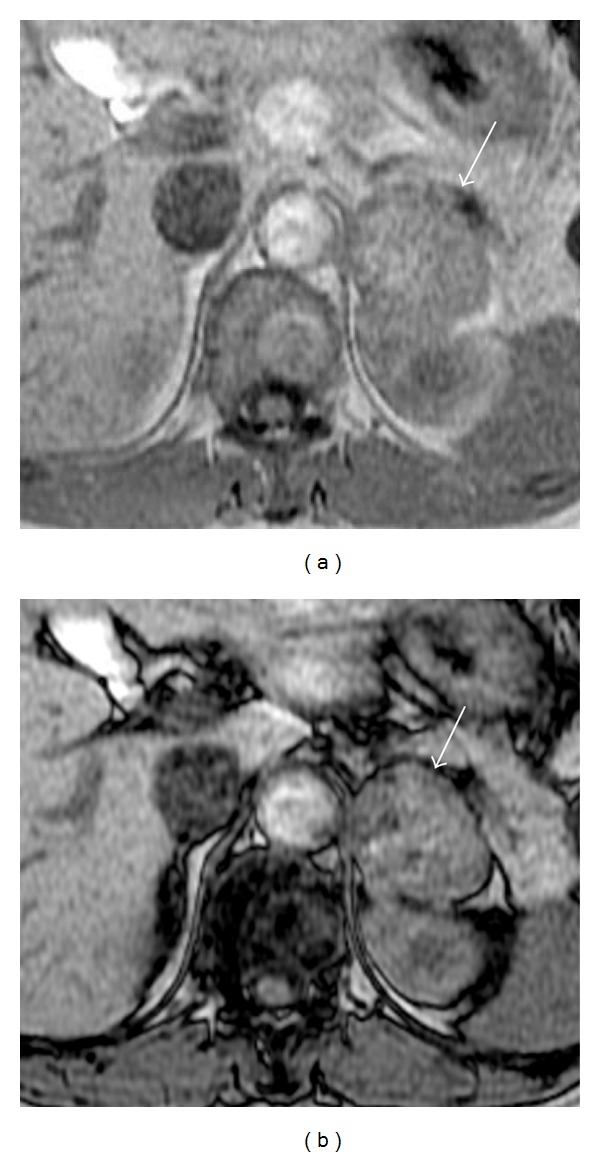
In-phase gradient echo MRI demonstrated a 40 mm, well-defined left adrenal mass (a) (arrow). Opposed-phase imaging showed signal decrease in part of the mass (b), indicating lipid content.

A 48-year-old woman with hypertension underwent CT for her gluteal pain. A left adrenal mass was found, and she was referred to our hospital to evaluate the mass. Magnetic resonance (MR) imaging showed a 40 mm, well-defined, left adrenal mass (Figure 1). In-phase and opposed-phase gradient echo images indicated lipid content in the mass. Adrenocortical adenoma was suggested, although the relatively large size also raised suspicion of adrenocortical carcinoma.

She was admitted for further evaluation. On admission, her blood-pressure was 180/110 mmHg, and she had swelling in her right leg. Endocrinological studies showed increased cortisol (22.7 pg/mL, normal 4.0–19.3 pg/mL), suppressed adrenocorticotropic hormone (ACTH; <2.0 pg/mL, normal 7.2–63.3 pg/mL), normal aldosterone (70.5 pg/mL, normal 39–307 pg/mL), increased serum dehydroepiandrosterone sulfate (697 pg/mL, normal 33–262 pg/mL), and normal serum catecholamine levels (noradrenaline 71 pg/mL, normal 100–450 pg/mL; adrenaline 9 pg/mL, normal <100 pg/mL; dopamine <5 pg/mL, normal <20 pg/mL). Preclinical Cushing's syndrome was considered based on these results.

**Figure 2:**
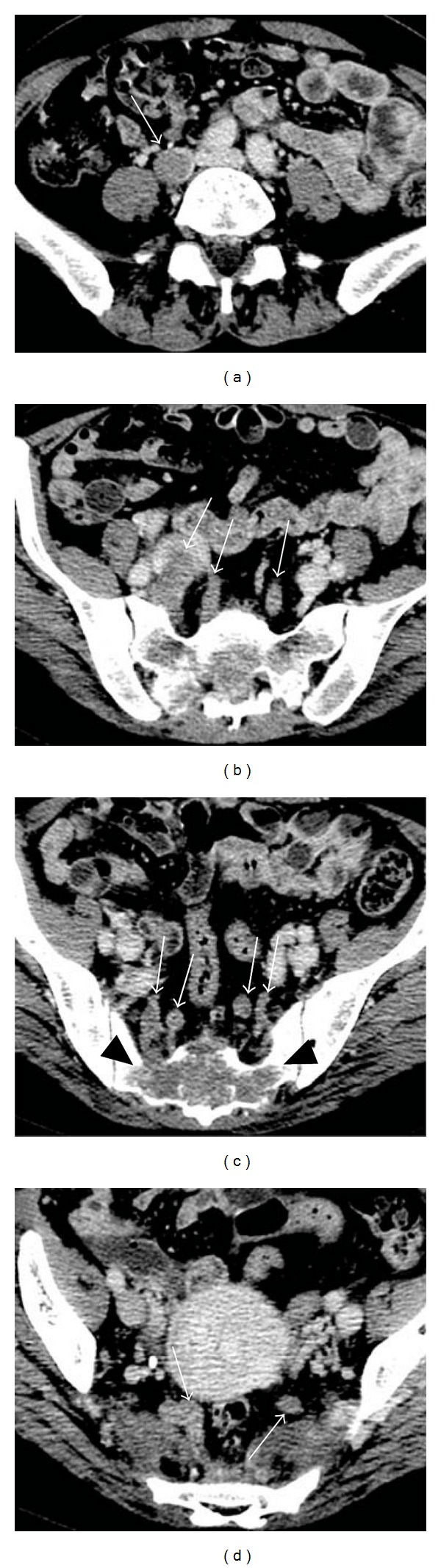
Contrast-enhanced CT demonstrated intraluminal filling defects in the right common iliac vein, both internal iliac veins, and their branches ((a)–(d)) (white arrows). A lytic lesion in the sacrum was also noted (c) (black arrowheads) and continuity between the sacral lesion and the filling defect in the branch of the right internal iliac vein was indicated.

She was suspected of having deep venous thrombosis because of an elevated d-dimer level (9.44 *μ*g/mL, normal <1.0 *μ*g/mL) and swelling of the right leg. Ultrasound evaluation of her lower extremities showed a hyperechoic area and interrupted intraluminal blood flow in the right common iliac vein, consistent with venous thrombosis. Contrast-enhanced CT demonstrated filling defects in the left pulmonary artery, suggestive of pulmonary thromboembolism, in addition to the left adrenal mass. Moreover, lytic changes were seen in the sacral bone, and intraluminal filling defects were noted in the branches of the right internal iliac vein, presumably in contact with the sacral bone lesion (Figure 2). The filling defects reached the upper portion of the right common iliac vein. Intraluminal filling defects were also noted in the left internal iliac vein and its branches. These findings suggested tumor thrombosis extending from a sacral bone metastasis of adrenocortical carcinoma.

**Figure 3:**
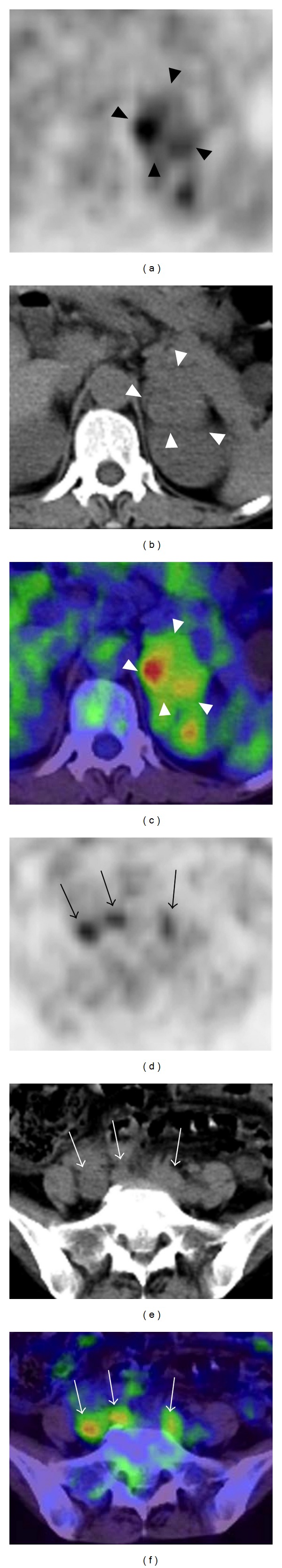
Axial PET (a), CT (b), and PET-CT fusion (c) images showed increased FDG uptake in the left adrenal mass (arrowheads). Increased uptake was also seen in the pelvic veins, consistent with a tumor thrombus ((d)–(f)) (arrows).

We inserted an inferior vena cava filter via the left femoral vein and obtained a small sample of the thrombus from the upper part of the right common iliac vein. Histopathology confirmed tumor thrombosis and indicated the metastasis of renal cell carcinoma or adrenocortical carcinoma. ^18^F-FDG PET-CT was performed for whole-body evaluation of malignant spread and showed increased uptake in the left adrenal gland and sacral bone, consistent with the malignant nature of the known lesions (Figure 3). It also demonstrated increased uptake in front of the sacral bone, corresponding to the distribution of thrombotic lesions shown on contrast-enhanced CT. This finding was considered to favor the diagnosis of tumor thrombosis over venous blood thrombosis. No other focus of abnormal ^18^F-FDG uptake was noted.

**Figure 4:**
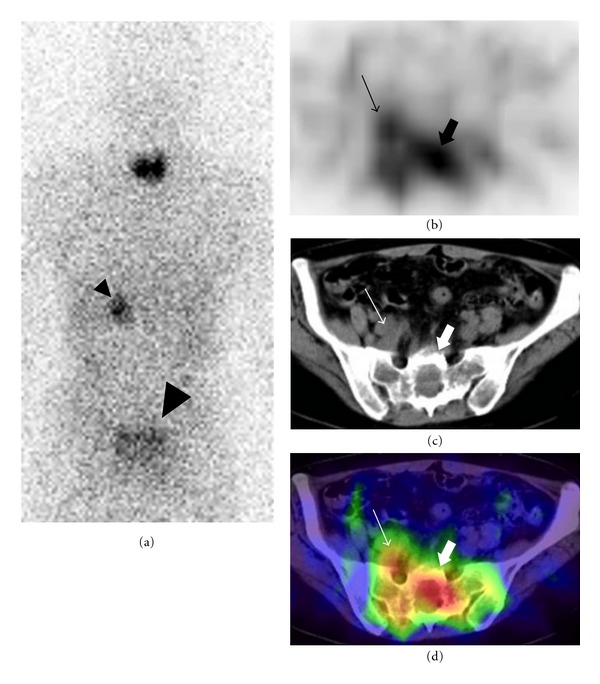
The posterior whole-body adrenocortical scintigraphy image showed focal uptake in the left adrenal (small arrowhead) and pelvic (large arrowhead) lesions (a). The axial SPECT (b), CT (c), and fused SPECT-CT (d) images confirmed increased uptake in the sacrum (thick arrows) and indicated increased uptake in the vein (thin arrows).

A CT-guided biopsy of the sacral bone lesion was performed to confirm that it was an adrenocortical carcinoma metastasis. Then, adrenocortical scintigraphy was performed for functional evaluation of the left adrenal tumor and pelvic lesions. Anterior and posterior planar images obtained 10 days after an intravenous injection of 18.5 MBq ^131^I-iodomethylnorcholesterol showed high uptake in the left upper abdomen and pelvis. Subsequent SPECT-CT demonstrated that the pelvic uptake was located mainly in the sacral bone and extended anteriorly (Figure 4), presumably corresponding to the venous thrombus. The adrenocortical scintigraphy findings supported that the left adrenal mass, sacral bone lesion, and venous thrombus were all likely of adrenocortical origin.

The pathology of the sample taken from the sacral bone lesion resembled that of the pelvic venous thrombus and indicated renal cell carcinoma or adrenocortical carcinoma. Considering the lack of abnormalities in the kidneys on various images, together with the pathology diagnosis, a final diagnosis of adrenocortical carcinoma with pelvic venous tumor thrombosis extending from sacral bone metastasis was made. Subsequently, chemotherapy was started and she died 1 year after the final diagnosis.

## 3. Discussion

Adrenocortical carcinoma can cause venous tumor thrombosis in the inferior vena cava, portal vein, and renal vein [1], and extensive tumor thrombosis reaching the right atrium has been reported [2, 3]. In our patient, contrast-enhanced CT showed extensive filling defects in the pelvic veins that were histopathologically diagnosed to represent tumor thrombosis. The tumor thrombus was far from the primary adrenocortical carcinoma and adjacent to the sacral bone metastasis, indicating venous invasion from the sacral bone metastasis and subsequent extension in the venous system. It is inferred that adrenocortical carcinoma can grow within the veins preferentially.

In our patient, ^18^F-FDG PET revealed extensive increased uptake corresponding to the filling defects in the pelvic veins demonstrated on CT. Although intense ^18^F-FDG uptake has been reported in a benign thrombus [4], high ^18^F-FDG accumulation in a thrombus generally indicates a tumor thrombus [5, 6]. Tumor thrombosis can cause blood thrombosis via insufficient circulation. The extensive ^18^F-FDG uptake in our patient appeared to support extensive growth of the tumor thrombus itself.

It has been reported that a metastatic lesion of adrenocortical carcinoma can be visualized as a hot spot on adrenocortical scintigraphy with ^131^I-iodomethylnorcholesterol [7–9]. In our patient, adrenocortical scintigraphy demonstrated increased uptake in both the left adrenal and sacral lesions. Although false-positive increased uptake in a renal cell carcinoma metastasis has been reported [10], increased uptake on adrenocortical scintigraphy implied that the sacral lesion originated from adrenocortical tissues and was an adrenocortical carcinoma metastasis.

The aid of accurate coregistration improves the localization of radiotracer uptake with CT PET-CT and SPECT-CT. Although SPECT-CT is not commonly performed for adrenocortical scintigraphy, in our patient, SPECT-CT increased the confidence that the high uptake in the pelvis was in the osteolytic lesion in the sacrum. Furthermore, it showed anterior extension of the high uptake from the sacral lesion, which suggested uptake in the venous thrombus and was consistent with the diagnosis of a tumor thrombus originating from the adrenocortical carcinoma. It is difficult to obtain high-quality SPECT images with ^131^I-iodomethylnorcholesterol because of the low injection dose and high-energy gamma rays from ^131^I. However, fusion with CT facilitated detailed interpretation of the low-quality SPECT images and SPECT-CT provided additional information in adrenocortical scintigraphy.

In summary, we report the imaging findings of a patient with an adrenocortical carcinoma who had a pelvic tumor thrombus extending from a sacral bone metastasis. Multimodal imaging including ^18^F-FDG PET-CT and SPECT-CT might be beneficial for the characterization and localization of lesions in patients suspected of having metastatic adrenocortical carcinoma.
